# Investigating the Effects of Distillers Grains on Heifer Feeding Behavior in the Finishing Phase

**DOI:** 10.3390/ani11071905

**Published:** 2021-06-26

**Authors:** Kaylie A. Huizenga, Joshua C. McCann

**Affiliations:** Department of Animal Sciences, University of Illinois, Urbana, IL 61801, USA; kaylieh2@illinois.edu

**Keywords:** distillers grains, feeding behavior, feed behavior variation, feedlot

## Abstract

**Simple Summary:**

Dried distillers grains (DDG), a corn by-product of the ethanol industry, are a common feedstuff in cattle diets. Distillers can be used in diets as an effective source of protein and can reduce risk of acidosis by reducing the highly fermentable carbohydrate of starch. However, the price and availability of distillers grains are variable based on droughts, energy prices, and other factors. When inclusion of distillers grains in diets becomes uneconomical or is not possible, diets may decrease in crude protein and increase in starch-altering rumen fermentation parameters and feeding behavior. The effect of distillers grains on cattle growth performance and carcass traits has been studied extensively, but little is known about how distillers inclusion affects feeding behavior. Therefore, the objective of this experiment was to determine the effect of low inclusion levels of DDG on feeding behavior in heifers consuming a high-moisture corn-based diet in the finishing phase. This study demonstrated that low inclusion levels of distillers grains has little effect on growth performance, but can alter feeding behavior and reduce variability in feeding behavior traits.

**Abstract:**

The objective was to determine the effect of low inclusion levels of dried distillers grains (DDG) on feeding behavior in heifers consuming a high-moisture corn-based diet in the finishing phase. Simmental × Angus heifers (N = 90; 323 ± 50 kg) were fed for 180 d. Heifers were blocked by initial body weight (BW) into two groups, stratified by sire, and assigned to 15 pens with six heifers each. Pens were randomly assigned to one of three dietary treatments: 0% DDG inclusion (0DG), 7% DDG inclusion (7DG), or 14% DDG inclusion (14DG). Treatments did not affect (*p* > 0.59) BW, average daily gain, and gain:feed. Although there was a treatment × time effect (*p* = 0.05) for dry matter intake (DMI), with 0DG having greater DMI during the last 70 d, no differences in overall DMI were detected. Treatment affected (*p* < 0.01) bunk visit duration and head down duration, with 7DG and 14DG having less minutes per day. Bunk visit frequency (*p* = 0.02) was less variable for heifers fed 14DG and DMI tended (*p* = 0.08) to be less variable for both distillers treatments. While dietary inclusion of DDG has minimal effects on overall heifer performance, low levels of DDG inclusion can affect feeding behavior and intake variation.

## 1. Introduction

Corn is the primary feedstock used to produce ethanol in North America through the dry milling process, which results in a coproduct known as dried distillers grains (DDG). During the distilling process, starch is removed from corn through yeast fermentation, resulting in a coproduct that is more concentrated in fat, fiber, and protein levels [1]. Dried distillers grains, a common feedstuff in cattle diets, have been used as an effective protein source, while reducing acidosis by reducing the highly fermentable carbohydrate of starch. However, DDG availability can become an issue as prices are susceptible to droughts, energy prices, and other factors [2]. In the spring of 2020, the availability of DDG was significantly decreased due to the impact of the COVID-19 outbreak. Pandemic travel restrictions and implementation of the Phase One trade deal caused a decline in ethanol production and DDG [3]. Without cost-effective protein sources such as DDG, protein levels in diets can be reduced to less than 12.5%. Decreased DDG also typically translates to greater corn inclusions, which increases dietary starch and acidosis risk. Cattle are also being fed to heavier weights [4] with more days on feed compared with the last time diets with little to no DDG were fed in the 1990s [1]. Starch inclusion levels can affect rumen function, digestibility, and, overall, change feeding behavior [5]. The long-term effects distillers have on feeding behavior and variation in intake are yet to be evaluated. Therefore, the objective of this study was to determine the effect of low inclusion levels of DDG on feeding behavior in heifers consuming a high-moisture corn-based diet in the finishing phase.

## 2. Materials and Methods

Experimental animals were managed according to the guidelines recommended in the Guide for the Care and Use of Agricultural Animal in Agricultural Research and Teaching (Federation of Animal Science Societies, 2010). All experimental procedures were approved by the Institutional Animal Care and Use Committee of the University of Illinois (IACUC #17292). 

### 2.1. Experiment Design, Animals, and Management

Ninety Simmental × Angus heifers were utilized for the current study. The experiment was conducted as a randomized block design. Heifers were blocked by initial body weight (BW) into 2 groups, stratified by sire, and assigned to 15 pens with 6 heifers/pen. Pens were randomly assigned to 1 of 3 dietary treatments (Table 1): (1) 0% dried DDG inclusion (0DG), (2) 7% DDG inclusion (7DG), or (3) 14% DDG inclusion (14DG). Dried distillers grains were replaced with high-moisture corn, soybean meal, and urea (Table 1). Dietary crude protein (CP) ranged from 12.33 to 13.45% as CP decreased slightly, with lesser DDG to reflect typical changes observed in the feedlot industry. Pens (4.88 × 9.76 m) had concrete slats covered with rubber mats. During the adaptation phase, heifers were transitioned using the same diet and fed in concrete bunks for 17 d. Heifers were then fed in GrowSafe bunks (GrowSafe Systems Ltd., Airdrie, AB, Canada) to determine individual feed intake and feeding behavior. Heifers were transitioned to a new crop source of high moisture corn on d 102–104 and were fully transitioned to new high moisture corn on d 105.

Heifers were weighed on consecutive days upon start of the trial and before slaughter. Individual weigh dates were recorded on d 57 and 127. A 4% pencil shrink was applied to all weights to account for gut fill. Heifers were implanted 36 d prior to the start of the trial with a Component TE-IH with Tylan implant (80 mg trenbolone acetate, 8 mg estradiol USP, and 29 mg tylosin tartrate; Elanco) and on d 62 with a Component TE-200 with Tylan implant (200 mg trenbolone acetate, 20 mg estradiol USP, and 29 mg tylosin tartrate; Elanco). Heifers were fed Optaflexx 45 (ractopamine hydrochloride, Elanco) at 300 mg per heifer per day the last 28 days of the trial. On d 181, heifers were transported approximately 300 km to a commercial abattoir and were humanely slaughtered under USDA inspection. Hot carcass weight (HCW) was recorded immediately postharvest. Individual camera data were provided by Tyson Foods Inc. for determination of yield (formula derived from USDA, 1997) and quality grades.

All heifers were fed in concrete bunks prior to the trial, then switched to GrowSafe bunks at the start of the trial for determination of individual feed intake feeding behavior. Feeding behaviors and intake were monitored continuously in the GrowSafe system. Data were removed on individual days if less than 85% of the consumed feed was assigned to heifers or if the bunk was empty for more than 12 h. Meal criterion and meal analysis for each animal were determined using the Meal Criterion Calculator software version 1.8.7154.27227 (MCC; http://nutritionmodels.tamu.edu (accessed on 30 March 2021), verified 3 August 2019). Meal criterion is defined as the minimum non-feeding event interval between bunk visits before the next bunk visit is considered part of a new meal [6]. Uninterrupted bunk visit events are defined as back-to-back bunk visits for an animal at the same feed bunk [6]. Feed behavior variability was determined by calculating the standard deviation of each feed behavior trait for an individual animal for the entire experiment.

### 2.2. Sampling and Analytical Procedures

Feed ingredient samples were collected biweekly throughout the trial. Equal portions of each ingredient in each period were composited. Composites were dried in a 55 °C oven and ground through a Wiley mill (1-mm screen, Arthur H. Thomas, Philadelphia, PA, USA). Composites were analyzed for dry matter (DM; 24 h at 105 °C), neutral detergent fiber (NDF) and acid detergent fiber (ADF; using Ankom Technology method 5 and 6, respectively; Ankom^200^ Fiber Analyzer, Ankom Technology, Macedon, NY, USA), crude protein (CP; Leco TruMac, LECO Corporation, St. Joseph, MI, USA), ether extract (EE, Ankom method 2; Ankom Technology), and organic matter (OM; 600 °C for 12 h; Thermolyte muffle oven Model F30420C; Thermo Scientific, Waltham, MA, USA). 

**Table 1:** Diet composition and nutritional value.

Item	0DG	7DG	14DG
Ingredient, % DM inclusion			
High moisture corn	69.58	63.86	58.14
Corn Silage	13.34	13.34	13.34
Soybean Meal	2.86	1.43	--
DDGS ^1^	--	7.15	14.30
Supplement			
Ground corn	6.906	6.925	6.940
Limestone	1.477	1.620	1.763
Urea	0.953	0.791	0.634
Trace mineral premix ^2^	0.097	0.097	0.097
Rumensin 90	0.016	0.016	0.016
Fat	0.080	0.080	0.080
Supplement			
MGA ^3^	0.013	0.013	0.013
Ground corn	4.677	4.677	4.677
Chemical analysis, % DM			
Dry matter	65.28	65.94	66.70
Organic matter	92.34	92.09	91.92
Crude protein	12.33	12.85	13.45
Neutral detergent fiber	11.20	12.46	13.74
Acid detergent fiber	5.54	5.81	6.09
Ether extract	3.34	3.88	4.42
NE_m_^4^, Mcal/kg	1.98	1.98	1.98
NE_g_^4^, Mcal/kg	1.46	1.46	1.46

^1^ Dried distillers grains plus solubles; ^2^ Supplement contained ^2^ 8.5% Ca, 5% Mg, 7.6% K, 6.7% Cl, 10% S, 0.5% Cu, 2% Fe, 3% Mn, 3% Zn, 278 mg/kg Co, 250 mg/kg I, 150 mg/kg Se, 2205 KIU/kg Vit A, 662.5 KIU/kg Vit D, 22,047.5 IU/kg Vit E. ^3^ MGA 200, Zoetis, Parsippany, NJ; ^4^ Calculated net energy for maintenance (NE_m_) and net energy for gain (NE_g_) values from National Academies of Sciences, Engineering, and Medicine [7].

### 2.3. Statistical Analysis

The experimental design was a randomized block with pen as the experimental unit. The MIXED procedure of SAS 9.4 (SAS Inst. Inc., Cary, NC, USA) was used for all statistical analysis. The model included fixed effects of block, diet, time, and the interaction of diet and time. Pen nested in the treatment was included as a random effect [8]. Start BW and the most appropriate expected progeny differences (EPD) estimate were used as a covariate for BW, ADG, G:F, DMI, and all carcass traits to help account for initial and inherent genetic differences. When there was not an appropriate EPD, sire was included as a fixed effect. A repeated measures statement was used for time effect to analyze BW, ADG, G:F, and DMI using an autoregressive (1) covariate structure based on model fit statistics. When analyzing variation in feeding behavior traits, individual animal average for the feed behavior was used as a covariate. Cook’s D was used to identify outliers. When residuals were not normally distributed for several feeding behavior traits, the Box–Cox procedure was used to determine the appropriate transformation. Least squares means have been back-transformed for ease of interpretation. Significance was declared at *p* ≤ 0.05 and tendencies were discussed at 0.05 < *p* ≤ 0.10.

## 3. Results

### 3.1. Growth Performance 

Overall, BW, ADG, and G:F (Table 2) did not have a diet × time or diet effect (*p* ≥ 0.11). Although, DMI was also not affected by diet (*p* = 0.29), there was a diet × time effect observed (*p* < 0.05). On d 56–126, there was a tendency (*p* = 0.09) for heifers fed 0DG to have a greater DMI when compared with 7DG and 14DG. On d 126–180, heifers fed 0DG also had a greater (*p* < 0.01) DMI than 7DG and 14DG. 

### 3.2. Feeding Behavior

Feeding behavior traits including bunk visit duration and head down did have a diet effect (*p* < 0.01; Table 3). Heifers fed 0DG had increased bunk visit durations and head down duration compared with 7DG and 14DG. Heifers fed 14DG had less variation (*p* = 0.02) in their bunk visit frequency throughout the trial. Both diets including DDG also had a tendency (*p* = 0.08) to have decreased variation in DMI. Heifers fed 14DG also had a tendency (*p* = 0.06) to have less variation in meal frequency. However, heifers fed 0DG had a tendency (*p* = 0.07) to have less variation in meal duration. There was no diet effect (*p* ≥ 0.72) for meal frequency, meal duration, meal length, meal size, and eating rate. There was also no diet effect (*p* ≥ 0.13) for variation in behavior traits, including bunk visit duration and head down duration. 

### 3.3. Carcass Traits

Carcass data (Table 4) reveal that heifers fed 7DG had smaller (*p* = 0.02) longissimus muscle (LM) area. No other diet effects were present (*p* ≥ 0.23) for hot carcass weight, dressing percentage, 12th rib fat thickness, yield grade, marbling, or kidney, pelvic, or heart fat. 

## 4. Discussion

The availability and price of coproducts such as DDG are a primary factor driving inclusion in feedlot diets. Determining how to optimally use DDG to alter feeding behavior and feed intake variation has not been studied extensively. In contrast, its effect on growth and performance is well-known. In the current study, no heifer overall performance advantages were observed with the low inclusion levels of DDG. Similar to the current study, ADG and G:F were not different for heifers consuming diets with 0% and 13% DDG [9]. When increasing DDG inclusion up to 40%, a quadratic ADG response was observed with the greatest ADG at a 20% inclusion [10]. However, most distillers titration studies substitute DDG with corn and urea and also use diets with greater crude protein levels than the current study. Corn processing methods may also affect growth results. When comparing the use of high moisture corn and rolled corn in distillers diets, ADG is increased and G:F is improved for cattle fed high-moisture corn [11]. When substituting DDG with high-moisture corn, soybean meal, and urea, inclusions of DDG at 15% or less may not offer any performance advantages.

A meta-analysis [1] demonstrated that increasing DDG up to 40% resulted in a quadratic effect for DMI, with intake maximized at 30% DDG inclusion. A direct comparison of 0 and 15% DDG inclusions indicated no difference in overall DMI [12]. However, the 15% DDG diet did increase DMI during the initial 28 d of the finishing phase [12]. In contrast, DMI was increased for heifers fed 0DG in the final 70 d of the current study. Most studies, including those in the meta-analysis [1], only analyze DMI for the whole feeding period rather than in time intervals. Therefore, it is difficult to compare the current findings with the existing literature on how DMI is affected throughout the finishing phase.

Despite the importance of coproducts in cattle feeding diets, little is known about how they alter feed intake behaviors. Feeding behaviors are important to understand feed intake regulation, feed efficiency, and health status [6]. Specifically, cattle with greater G:F spend less time at the bunk, have a greater eating rate, and have less variation in feed intake [13], which may play a part in feeding behavior responses to distillers inclusion levels due to decreased starch in the diet. When comparing diets without distillers to those with 16% DDG, distillers inclusion increased meal size and eating rate as cattle decreased the number of meals and time at bunk [14]. Importantly, these steers were only fed for 122 d, which was a shorter period of time compared with the current study. Although there were no differences in meal size or eating rate in the current experiment, there was a decrease in time spent at the bunk with inclusion of distillers similar to the described study. Crude protein in the diets of the study previously described was also much greater with 0% distillers inclusion at 16% CP due to the use of alfalfa mixed haylage as the forage source and the 0DG diet of the current study only having 12.33%. Steers were also used in the study previously described, whereas the current study used heifers. However, there is no current research evaluating feeding behavior differences between steers and heifers consuming DG. 

Variation in feed intake over time can be managed by several strategies and is an important factor for feedlot growth performance and efficiency [15]. However, variation in feed intake and behaviors is rarely reported. Decreasing DDG inclusion would typically lead to greater dietary starch and risk for acidotic bouts. Thus, it was hypothesized that 7DG and 14DG would have less variation in feed intake and feed behaviors during the finishing phase. Feed intake and variation in feed intake among days can be used to detect ruminal acidosis. Decreased DMI and increased variation in intake are often a sign of subacute acidosis [5]. As hypothesized, there was a tendency for DMI variation to be increased in heifers fed 0DG. Variation in bunk visit frequency duration was also decreased with heifers fed 7DG and 14DG. As with any evaluation of an ingredient such as DDG, the observed effects cannot be solely attributed to DDG inclusion because of a concomitant decrease in dietary starch. Thus, other strategies to reduce dietary starch may also decrease variation in DMI and feeding behaviors.

Previous research on carcass data has reported that DDG inclusions of 0–40% may not affect carcass traits. When comparing 20% to 40% distillers grain inclusion levels, HCW, LM area and marbling were not affected by inclusion levels [16]. Similarly, no differences between carcass characteristics in steers fed 0%, 15% or 25% DDG have been found in other studies [10]. Similar results were found in the current study except for LM area, which was decreased in heifers fed 7DG, despite final BW and HCW being similar and genetic differences being accounted for by the EPD for LM area. However, LM area reduction has also been observed and unexplained in other wheat and triticale distillers grains studies [17,18].

## 5. Conclusions

Heifers were fed different inclusion levels of DDG to evaluate the effect of low DDG inclusion on feeding behavior compared to a high starch control diet. There was no treatment effect for growth performance. While heifers fed 0DG had greater DMI on d 126–180 than 7DG and 14DG, there was no treatment effect on overall DMI. Feed intake behaviors were affected by treatment as bunk visit duration and head down duration were decreased for heifers fed 7DG and 14DG compared with 0DG. Overall, the results indicated that inclusion of distillers has minimal impact on overall performance and intake but affect some feeding behavior traits. Inclusion of DDG also decreased variation in bunk visit frequency and tended to decrease variation in DMI. Even at low dietary inclusion rates of 7% and 14% of the diet, DDG can modify and reduce variation in feeding behavior in feedlot heifers consuming a high-moisture corn-based diet. 

## Figures and Tables

**Table 2:** Effect of distillers grains inclusion level on body weight (BW), average daily gain (ADG), dry matter intake (DMI), and gain to feed ratio (G:F).

Item	0DG	7DG	14DG	SEM	*p*-Value		
					Diet	Time	Diet × Time
No. of pens	5	5	5				
BW, kg ^1^							
d 56	382	388	386	4.3	0.95	<0.01	0.44
d 126	487	488	485				
d 180	556	551	551				
ADG, kg/day							
d 0–56	1.34	1.45	1.44	1.552	0.85	<0.01	0.11
d 56–126	1.55	1.49	1.47				
d 126–180	1.35	1.23	1.27				
DMI, kg/day							
d 0–56	8.13	8.29	8.26	0.198	0.29	<0.01	0.05
d 56–126	9.30	8.83	8.82				
d 126–180	9.68 ^a^	9.09 ^b^	9.04 ^b^				
G:F							
d 0–56	0.164	0.176	0.174	0.004	0.59	<0.01	0.44
d 56–126	0.168	0.169	0.166				
d 126–180	0.138	0.136	0.140				

^a,b^ Means bearing different subscripts in same row are significantly different (*p* < 0.01).^1^ 4% pencil shrink was applied to all body weights.

**Table 3:** Effect of distillers inclusion on feeding behavior traits and the variability of each feeding behavior trait.

Items	0DG	7DG	14DG	*p*-Value
No. of pens	5	5	5	
Feeding behavior traits ^1^				
Meal frequency, events/d	15.1	14.0	13.7	0.88
Meal duration, min/d	134.9	126.5	108.5	0.68
Meal length, min	8.2	7.4	9.0	0.91
Meal size, kg	0.66	0.87	0.89	0.65
Meal eating rate, g/min	69.4	80.1	79.4	0.72
BV frequency, visit/d	30.2	28.5	26.9	0.12
BV duration, min/d	83.1 ^a^	71.4 ^b^	69.0 ^b^	<0.01
Head down, min/d	34.9 ^a^	26.5 ^b^	25.5 ^b^	<0.01
Feeding behavior variability ^2^				
DMI	4.6 ^a^	4.3 ^b^	4.2 ^b^	0.08
Meal frequency	3.6 ^ab^	4.0 ^a^	3.4 ^b^	0.06
Meal duration	44.4 ^b^	47.9 ^ab^	52.5 ^a^	0.07
BV frequency	10.2 ^a^	9.5 ^ab^	8.7 ^b^	0.02
BV duration	20.4	20.3	21.9	0.68
Head down	15.0	15.1	16.5	0.58

^a,b^ Means bearing different subscripts in same row are significantly different (*p* < 0.04). ^1^ Meal frequency = average number of meals each day; Meal duration = sum of lengths of meal events recorded each day; Meal length = average meal length; Meal size = average DM intake per meal; Meal eating rate = DM intake/meal duration; BV frequency = number of bunk visits (BV) recorded each day; BV duration = sum of the length of all BV events recorded each day; Head down = number of electronic radio frequency identification (RFID) reads each day multiplied by the scan rate of the GrowSafe System (GrowSafe Systems Ltd., Airdrie, AB, Canada). ^2^ Calculated from standard deviation of feeding behavior.

**Table 4:** Effect of distillers inclusion on carcass characteristics.

Items ^1^	0DG	7DG	14DG	SEM	*p*-Value
No. of pens	5	5	5		
HCW, kg	356	351	357	3.789	0.44
Dressing percentage, %	64.09	63.84	64.73	0.404	0.14
12th rib fat thickness, cm	1.45	1.60	1.61	0.073	0.23
LM area, cm^2^	91.00	83.69	89.70	0.826	0.02
KPH, %	2.18	2.20	2.15	0.037	0.62
Yield grade	3.09	3.34	3.19	0.103	0.23
Marbling score ^2^	584	562	546	19.8	0.39

^1^ HCW = hot carcass weight; LM area = longissimus muscle area; KPH = kidney pelvic heart fat; ^2^ 400 = Choice USDA Quality Grade, 500 = Average Choice USDA Quality Grade, 700 = Prime USDA Quality Grade.

## Data Availability

Data sharing is not applicable to this article.
